# Integration of Endodontic Services Into Preventive Oral Health Care: A Narrative Review

**DOI:** 10.1155/ijod/6859907

**Published:** 2026-06-26

**Authors:** Chitharanjan M. Shetty, Derek Shaji Pious, Maria Anna Geevarghis, Shreya Hegde, Rashi Shroff, Gurmeen Kaur

**Affiliations:** ^1^ Department of Conservative Dentistry and Endodontics, A. B. Shetty Memorial Institute of Dental Sciences, NITTE (Deemed to be University), Deralakatte, Mangalore, 575018, Karnataka, India, nitte.edu.in; ^2^ Department of Conservative Dentistry and Endodontics, Manipal College of Dental Sciences Mangalore, Manipal Academy of Higher Education, Manipal, India, manipal.edu; ^3^ Department of Conservative Dentistry and Endodontics, Sri Guru Ram Das Institute of Dental Sciences and Research, Amritsar, India, sgrddental.org

**Keywords:** endodontics, oral health, preventive dentistry, primary health care, regenerative endodontics, universal health care

## Abstract

**Background:**

The integration of endodontic services into preventive oral health care has become increasingly important as dental caries and pulpal pathologies remain major contributors to the global burden of oral disease. However, despite advances in preventive dentistry, gaps persist in the early identification and management of pulpal disease within primary care systems. Integrating endodontic services may enhance early diagnosis, timely intervention, and long‐term tooth preservation.

**Methods:**

A narrative review of published literature was conducted to examine current models, strategies, and evidence related to integrating endodontic care within preventive oral health frameworks. Key databases and professional organizational documents were reviewed to identify themes including service delivery models, workforce considerations, recent technological advances, barriers, and policy directions. Methodological rigor was enhanced using structured narrative review approaches and guided thematic synthesis.

**Results:**

Evidence indicates that integration enhances continuity of care, improves access to early pulp therapy, and reduces disease progression. Successful models include referral‐based coordination, colocation of care, and deployment of practitioners with enhanced endodontic skills. Technological developments such as digital diagnostics, artificial intelligence, and minimally invasive endodontic techniques further support integration. However, barriers such as fragmented health systems, limited workforce capacity, inconsistent funding, and inadequate training persist. Importantly, the current evidence base remains heterogeneous, with limited long‐term outcome data and variability in implementation across health systems. Policy frameworks and interprofessional collaboration are essential to overcoming these challenges.

**Conclusion:**

Integrating endodontic services into preventive oral health systems is feasible and beneficial, offering improved patient outcomes and stronger primary care delivery. Adoption of standardized referral pathways, workforce development, digital infrastructure, and supportive policies can facilitate broader implementation. Future research should prioritize cost‐effectiveness analyses, standardized outcome measures, and evaluation of long‐term clinical impact to support scalable integration models.

## 1. Introduction

Dental diseases, particularly caries and pulpal disorders, continue to represent a major challenge for global health systems, significantly contributing to pain, infection, tooth loss, and reduced quality of life. Although preventive strategies have significantly reduced the burden of early‐stage disease, progression to pulpal pathology remains common, highlighting a gap in timely intervention within primary care systems. Incorporating endodontic care into preventive oral health strategies may address this gap by enabling earlier identification of pulpal involvement and facilitating timely therapeutic intervention. Evidence from existing integrated oral health models indicates that coordinated approaches to service delivery enhance accessibility, minimize delays in treatment, and promote comprehensive, patient‐centered management [1–3].

Contemporary public health perspectives increasingly recognize that endodontic care should not be viewed in isolation but rather as an integral element of prevention‐oriented oral health systems [4, 5]. International organizations, including the FDI World Dental Federation, along with various national authorities, underscore the importance of integration in achieving equitable access to care and advancing minimally invasive and preventive treatment philosophies [6–8]. When effectively implemented, such integrated approaches are associated with improved clinical outcomes, greater patient satisfaction, and more efficient utilization of healthcare resources [9–11].

Despite these advantages, the implementation of integrated endodontic care is often constrained by systemic challenges. These include shortages in trained personnel, lack of standardized referral mechanisms, fragmentation within public dental services, and variability in clinical expertise among providers [12–14]. These limitations indicate that integration is not merely a clinical consideration but a system‐level challenge requiring coordinated policy, workforce, and technological solutions [15–18].

In this context, the present narrative review aims to critically synthesize current evidence on the integration of endodontic services within preventive oral health care systems, with a focus on service delivery models, workforce strategies, technological enablers, and policy frameworks. Additionally, this review seeks to identify existing gaps in the literature and propose a structured approach to guide implementation within diverse healthcare settings.

## 2. Methods

A narrative review is a comprehensive, critical, and objective analysis of the existing body of knowledge on a given topic, aimed at understanding key problem areas, theoretical frameworks, and the broader research context. This review was conducted in accordance with established narrative review methodologies and guided by the Scale for the Assessment of Narrative Review Articles (SANRA) to enhance methodological rigor and transparency.

The literature review was conducted using multiple bibliographic databases, including Medline, PubMed, Web of Science, Ovid, Scopus, ScienceDirect, and the Cochrane Library. In addition, gray literature and policy documents were identified through targeted internet searches using Google and Google Scholar, as well as publications and reports from international and national professional organizations, public health agencies, and oral health policy bodies.

### 2.1. The Literature Search Was Conducted in Two Stages

In the first stage, broad search terms were used to capture the overall scope of the topic. These included combinations of keywords such as endodontic services, preventive oral health care, primary dental care, integrated oral health care, pulpal disease, referral pathways, workforce models, and minimally invasive endodontics. Results from this stage were screened to identify recurring concepts and key thematic areas relevant to integration within preventive oral health frameworks.

In the second stage, a more focused literature search was undertaken based on the themes identified during the initial screening. These themes included the burden of endodontic and pulpal diseases, the role of early intervention within preventive oral health systems, service delivery and referral models, workforce and training considerations, digital and technological enablers, systemic and financial barriers, and policy frameworks supporting integrated endodontic care. Reference lists of selected articles were also hand‐searched to identify additional relevant publications.

The thematic domains identified during the first‐stage screening were subsequently used to refine the eligibility criteria for full‐text assessment. Studies were selected for inclusion if they contributed evidence relevant to one or more of the identified thematic areas, including service delivery integration, referral coordination, workforce models, preventive endodontic approaches, digital technologies, or policy frameworks related to integrated oral healthcare. This iterative approach ensured that the final included studies aligned closely with the conceptual objectives of the review while maintaining relevance to preventive oral health systems.

The search strategy incorporated both broad and specific keywords. Terms related to preventive oral health care included “fluoride,” “pit and fissure sealants,” “caries risk assessment,” and “oral health promotion.” Keywords related to minimally invasive endodontics included “vital pulp therapy,” “selective caries removal,” “indirect pulp capping,” and “pulp preservation.” These were combined with broader terms such as “endodontic care,” “primary dental care,” and “integrated care” using Boolean operators.

Although this is a narrative review, elements of structured reporting were informed by PRISMA 2020 guidelines to improve transparency; however, formal PRISMA or PRISMA‐ScR protocols were not strictly followed.

### 2.2. Search Strategy

A comprehensive literature search was conducted using electronic databases, including PubMed/MEDLINE, Scopus, Web of Science, Ovid, ScienceDirect, and the Cochrane Library. The search strategy combined Medical Subject Headings (MeSH) terms and free‐text keywords using Boolean operators.

The primary search terms included are as follows:

• “endodontic services” OR “endodontic care.”
• AND “preventive oral health care” OR “preventive dentistry.”
• AND “primary dental care” OR “primary health care.”
• AND “integration” OR “integrated care.”
• AND “pulpal disease” OR “root canal treatment.”

Additional terms such as “teledentistry,” “digital health,” “minimally invasive endodontics,” and “referral pathways” were included to capture emerging themes, with Boolean operators used to refine search combinations.

### 2.3. Time Frame of Literature Search

The literature search included studies published from 2000 to 2024, with an emphasis on recent developments to capture contemporary models of integrated endodontic care.

### 2.4. Study Selection Summary

**Figure 1:**
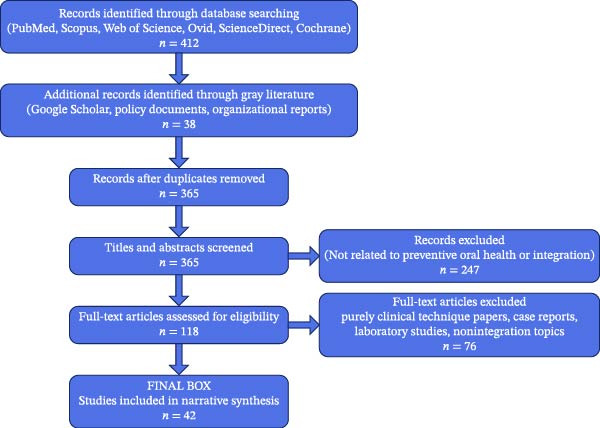
Flow diagram illustrating literature search, screening, eligibility assessment, and inclusion of studies for the narrative review.

The initial database search yielded 412 records across multiple databases, with an additional 38 records identified through gray literature sources. After the removal of duplicates, 365 records remained for title and abstract screening. Following screening, 118 articles were assessed for full‐text eligibility. Based on predefined thematic relevance and eligibility criteria, 42 studies were included for qualitative synthesis. A flow diagram illustrating the study selection process is presented in Figure 1.

### 2.5. The Review Applied the Following Inclusion and Exclusion Criteria

#### 2.5.1. Inclusion Criteria

• Studies addressing the integration of endodontic care within preventive or primary oral health systems.
• Studies discussing service delivery models, referral pathways, or workforce roles.
• Research related to minimally invasive or preventive endodontic approaches.
• Policy reports and reviews relevant to integrated oral healthcare.

#### 2.5.2. Exclusion Criteria

• Studies focused solely on advanced technical endodontic procedures without relevance to primary care.
• Laboratory‐based or in vitro studies.
• Case reports and opinion articles lacking broader clinical or public health relevance.
• Studies not addressing integration within oral health systems.

## 3. Results

The literature synthesis identified eight key thematic domains underpinning the integration of endodontic services within preventive oral health care systems.

1. Burden of endodontic and pulpal disease within preventive oral health systems.
2. Need for early endodontic assessment and intervention in primary oral health care.
3. Integration of endodontic services within preventive and primary care frameworks.
4. Service delivery models for integrated endodontic care, including referral‐based coordination and colocation of services.
5. Workforce capacity, training needs, and the role of enhanced‐skill practitioners in endodontics.
6. Role of minimally invasive and preventive endodontic approaches in tooth preservation.
7. Digital health and technological enablers supporting integration, including teledentistry and artificial intelligence.
8. Systemic, financial, and policy‐related barriers to the integration of endodontic services.

These themes were subsequently analyzed to explore patterns across health systems, identify common facilitators and barriers, and inform the development of a structured framework for integrated endodontic care.

## 4. Rationale for Integration

The identified literature was synthesized into thematic domains to contextualize the need for integrating endodontic services within preventive oral health systems. Evidence from national and international health systems with comparable workforce structures highlights key challenges and opportunities for integration.

### 4.1. Preventive Oral Health Care

Preventive oral health care focuses on the early identification and management of dental caries and other oral diseases before progression to advanced stages requiring complex interventions. Core strategies, including oral hygiene reinforcement, dietary counseling, caries risk assessment, and preventive interventions, play a crucial role in maintaining pulp vitality and reducing the need for endodontic treatment [1–3].

Fluoride plays a central role in preventive oral health care by enhancing remineralization, inhibiting demineralization, and exerting antibacterial effects on cariogenic biofilms. Its use in community‐based and clinical settings, such as fluoridated toothpaste, varnishes, and water fluoridation, has been shown to reduce the incidence and progression of early carious lesions. By arresting early‐stage caries, fluoride‐based interventions indirectly reduce the progression to pulpal involvement and the subsequent need for endodontic treatment. This highlights the critical link between preventive dentistry and the reduction of endodontic disease burden, reinforcing the rationale for integration [16, 19].

### 4.2. Burden of Endodontic Disease

Pulpal and periapical diseases represent a substantial portion of the global oral health burden and remain a leading cause of tooth pain, infection, and tooth loss [1]. Delayed identification and management of endodontic pathology at the primary care level contribute significantly to disease progression and increased treatment complexity. Early intervention can mitigate these outcomes and reduce the need for advanced procedures. Additionally, limited access to timely endodontic care in many public health systems exacerbates inequalities in oral health outcomes [3].

### 4.3. Need for Integrating Endodontic Services Into Preventive Care

Integrating endodontic care within preventive oral health programs supports continuity of care by linking disease detection, minimally invasive therapy, and timely referral or specialist intervention when needed [4–6]. Evidence suggests that integrated service delivery models improve coordination between providers, reduce fragmentation of care, and enhance overall health system efficiency [7, 8].

Incorporating endodontic competencies within primary care also facilitates early interventions such as vital pulp therapy, selective caries removal, and emergency pain management, which align with minimally invasive and prevention‐oriented principles [9–11].

### 4.4. Benefits of Integration

Integrating endodontic care into preventive oral health systems provides a wide range of clinical, organizational, and public health advantages. A primary benefit is the facilitation of early diagnosis and prompt intervention. Incorporating endodontic evaluation into routine preventive assessments enables the earlier identification of pulpal inflammation and reversible conditions [8].

This enables the use of conservative treatment approaches, such as vital pulp therapy and other minimally invasive techniques, thereby limiting progression to irreversible disease or apical pathology and enhancing long‐term tooth retention. Another important advantage is the improvement in continuity of care. Structured referral pathways and coordinated care models enable seamless transitions between preventive, primary, and specialist care.

These integrated frameworks help to shorten diagnostic and treatment timelines, reduce the likelihood of patients being lost to follow‐up, and ensure the timely completion of definitive care. Integration also enhances efficiency within the healthcare system by optimizing the use of the available resources. Approaches such as colocation of services and utilization of practitioners with enhanced endodontic skills improve resource allocation and reduce system inefficiencies [12].

Managing less complex cases within primary care while directing more challenging cases to specialists can decrease overall costs, shorten waiting times, and improve service productivity. From the patient’s standpoint, integrated endodontic care contributes to improved treatment experiences. Coordinated care pathways reduce delays, minimize the number of clinical visits, and improve clarity in communication regarding diagnosis and treatment planning. Structured follow‐up further supports adherence to treatment and reduces patient anxiety associated with fragmented care [1–3].

At a broader level, embedding endodontic services within preventive frameworks supports wider public health objectives. Early management of pulpal and periapical infections may reduce the systemic inflammatory burden associated with untreated oral disease. This aligns with the growing recognition of links between oral and systemic health and reinforces the role of oral healthcare within universal health coverage frameworks.

### 4.5. Conceptual Framework

**Figure 2:**
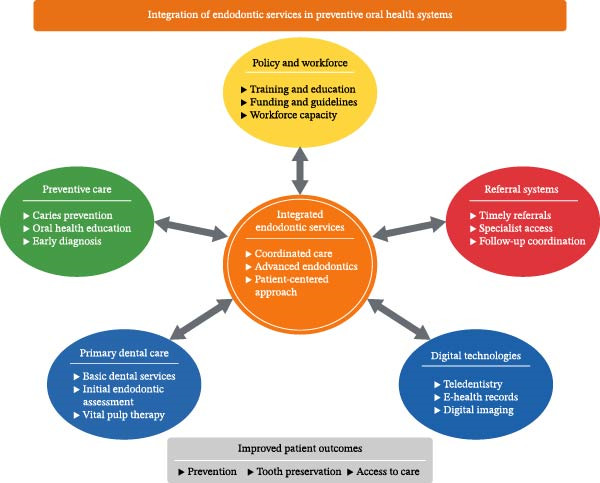
Conceptual framework illustrating the integration of endodontic services within preventive oral health systems. The model highlights the interaction between preventive care, primary dental care, referral pathways, digital technologies, and policy and workforce components, contributing to improved patient outcomes.

A conceptual framework for endodontic integration can be understood through three core pillars: service delivery alignment, ensuring primary care teams, enhanced‐skill practitioners, and specialists operate within coordinated pathways; workforce development expanding competencies, training, and task‐sharing to improve service coverage; and system enablers, including health policy support, digital infrastructure, and interprofessional collaboration [12–15]. These interconnected components collectively strengthen access, efficiency, and quality of endodontic care within preventive systems. Together, these elements support progressive integration from basic referral coordination to fully integrated service delivery models (Figure 2).

## 5. Models and Strategies for Integration (Figure 3)

Various models have been proposed to operationalize the integration of endodontic services within preventive oral health systems, each differing in complexity, resource requirements, and level of coordination.

**Figure 3:**
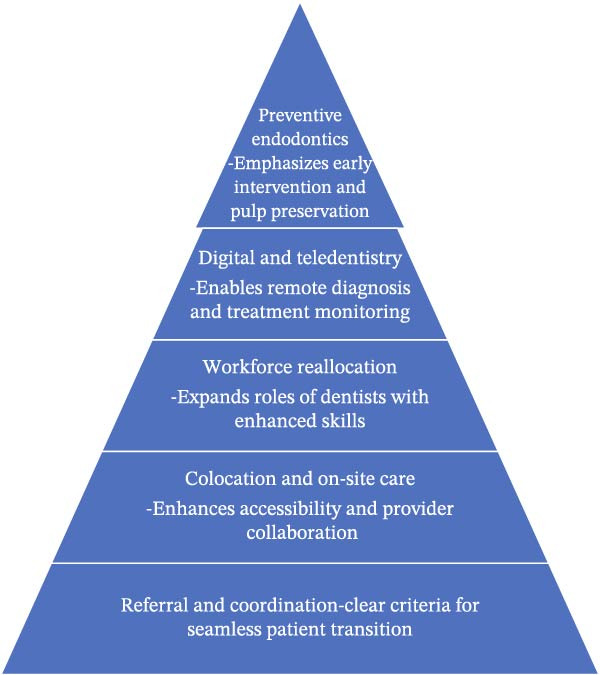
Endodontic integrated strategies.

### 5.1. Referral and Coordination

Referral‐based models remain the most widely implemented approach to integrating endodontic services within primary care systems. Effective implementation depends on clearly defined referral criteria, structured interprovider communication, and standardized workflows to ensure seamless patient transition from primary screening to endodontic intervention [13–15]. Such coordinated systems are associated with improved treatment timelines and more efficient utilization of public dental services; however, their effectiveness is highly dependent on system organization and adherence to referral protocols [16].

### 5.2. Colocation and On‐Site Endodontic Care

Colocation of endodontic services within primary care clinics enhances accessibility by reducing travel, minimizing appointment delays, and strengthening provider collaboration [17]. Models involving endodontists or enhanced‐skill practitioners operating within primary care settings have demonstrated improvements in continuity of care, reduced procedural backlogs, and enhanced patient satisfaction [18, 19]. However, implementation of colocation models may be limited by resource availability, infrastructure requirements, and workforce distribution, particularly in low‐resource settings.

### 5.3. Workforce and Task Reallocation

Expanding the roles of dentists with enhanced skills, alongside structured training for general practitioners, can improve the system capacity for delivering essential endodontic procedures [20–22]. Workforce reallocation strategies include the delegation of diagnostic responsibilities, provision of vital pulp therapy at the primary care level, and management of uncomplicated endodontic cases, thereby reducing the specialist burden and improving system efficiency [23]. The success of such models depends on standardized training, competency assessment, and clear clinical guidelines to ensure the quality and safety of care.

### 5.4. Digital and Teledentistry Integration

Digital systems support integration by enabling remote diagnosis, triage, referral validation, and monitoring of treatment outcomes [24]. Teleconsultation between general dentists and endodontists enhances diagnostic accuracy, facilitates prioritization of urgent cases, and reduces inappropriate referrals [23]. Digital imaging, electronic health records, and AI‐driven diagnostic tools further strengthen the communication and continuity of care across service levels. Despite these advantages, challenges such as infrastructure limitations, cost, and variability in digital literacy may affect widespread adoption.

### 5.5. Preventive Endodontics and Minimally Invasive Therapy

Preventive endodontics emphasizes early intervention, selective caries removal, and pulp‐preserving strategies to avoid disease progression and reduce the need for complex root canal treatment [19]. Minimally invasive techniques, including conservative access designs and contemporary instrumentation, enhance tissue preservation and clinical outcomes [22]. Integration of these approaches at the primary care level aligns with global preventive dentistry goals; however, successful implementation requires appropriate case selection and clinician expertise.

## 6. Evidence and Current Literature

The included literature demonstrates consistent trends supporting the integration of endodontic services within preventive oral health systems, particularly in improving access, efficiency, and patient outcomes across diverse healthcare settings.

### 6.1. Global Oral Health Burden

Global estimates consistently indicate that endodontic and pulpal diseases contribute substantially to the overall burden of oral conditions, particularly in low‐ and middle‐income populations [12]. Untreated dental caries frequently progresses to pulpal inflammation, creating a significant demand for urgent care that primary health systems often struggle to manage effectively [3]. Integration of endodontic competencies within preventive programs has been associated with reduced complications, fewer emergency visits, and improved long‐term tooth retention [4].

### 6.2. Integration in Primary Care Settings

Several studies highlight the successful integration of oral health services, including endodontics, into primary care settings [5–7]. These models consistently demonstrate improvements in accessibility, earlier disease detection, and more coordinated patient care pathways (Table 1). Key interventions, including shared‐care arrangements, deployment of enhanced‐skill practitioners, and structured referral pathways, are associated with higher treatment completion rates and improved patient satisfaction [8, 9].

**Table 1:** Summarizes the studies included in the qualitative synthesis relevant to integration of endodontic services within preventive oral healthcare systems.

Author (year)	Study design	Integration model	Key findings
Prasad et al. (2019) [1]	Systematic Review	Primary care integration	Improved access and early diagnosis
Harnagea et al. (2018) [2]	Scoping Review	Integrated care systems	Identified barriers and facilitators
Christian et al. (2023) [3]	Systematic Review	Preventive‐primary integration	Enhanced coordination and outcomes
Emami et al. (2016) [7]	Scoping Review	Primary care models	Improved accessibility and service delivery
Atchison et al. (2018) [8]	Policy Analysis	Integrated care frameworks	Highlighted implementation strategies
Melgaço‐Costa et al. (2016) [9]	Mixed‐methods	Public dental system	Improved patient satisfaction
Melgaço‐Costa et al. (2022) [10]	Observational	Public dental centers	Increased productivity
Al‐Haboubi et al. (2014) [11]	Observational	Enhanced‐skill dentists	Reduced waiting time
Gallagher and Wilson (2015) [12]	Workforce Study	DES model	Improved workforce capacity
Martins et al. (2016) [13]	Observational	Referral systems	Better coordination
Koch and Eriksson (2013) [15]	Program evaluation	General practice integration	Improved adoption
Wolters et al. (2017) [16]	Review	Minimally invasive endodontics	Improved outcomes
Duncan et al. (2023) [17]	Review	Regenerative endodontics	Future‐oriented care
Kaur et al. (2024) [18]	Review	AI integration	Improved diagnostics
van der Sluis et al. (2013) [19]	Review	Preventive endodontics	Early intervention benefits
Fullman et al. (2018) [20]	Global health study	UHC framework	Supports integration
Ghebreyesus (2017) [21]	Policy perspective	Health systems	UHC relevance
Peters et al. (2019) [6, 22]	Policy paper	FDI framework	Global integration strategy
ASTDD (2023) [4, 23]	Report	Primary care integration	Best practice models
Christian et al. (2024) [24]	Review	Future oral health systems	Emphasis on integration
Reit et al. (2007) [14]	Educational intervention	General dentist training	Improved adoption of rotary instrumentation
Hajizamani et al. (2012) [25]	Review	Primary healthcare integration	Supported integration into PHC systems
Crall (2005) [26]	Program development	Pediatric oral health integration	Improved coordination and referrals
Monajem (2006) [27]	Policy review	WHO oral health integration	Emphasized role of dental hygienists
Mumghamba (2014) [28]	Educational model	Workforce integration	Improved primary oral health training
Bourgeois et al. (2014) [29]	International collaborative study	Preventive oral health integration	Strengthened oral health education
Braun and Cusick (2016) [30]	Review	Medical–dental collaboration	Improved interdisciplinary coordination
Reddy et al. (2017) [31]	Review	Basic oral care package	Enhanced preventive oral healthcare
Blue and Riggs (2016) [32]	Review	Accountable care integration	Improved oral healthcare delivery
Jatrana and Crampton (2009) [33]	Policy analysis	Primary healthcare integration	Advocated oral health inclusion in PHC
Osazuwa‐Peters (2011) [34]	Health policy analysis	Primary oral healthcare	Identified limitations in oral healthcare delivery
Petersen et al. (2014) [35]	Policy review	Oral health systems strengthening	Reinforced PHC‐oriented oral health systems
Batra et al. (2014) [36]	Review	Basic package oral care	Promoted preventive oral healthcare models
Gauger et al. (2018) [37]	Scoping review	Collaborative care models	Improved pediatric oral health integration
Valentijn et al. (2013) [38]	Conceptual framework	Rainbow integration model	Provided integrated care framework
Sheiham and Watt (2000) [39]	Public health framework	Common risk factor approach	Linked oral and systemic disease prevention
Grover and Luthra (2013) [40]	Review	Oral‐systemic integration	Demonstrated diabetes–periodontitis relationship
Clarke and Braun (2018) [41]	Implementation framework	Primary care oral integration	Improved workflow integration
Sinaiko et al. (2017) [42]	Health systems analysis	Patient‐centered medical homes	Improved coordinated care
Basu and Bundick (2017) [43]	Health services research	Team‐based primary care	Strengthened proactive care models
Taguma and Barrera (2019) [44]	Policy report	Primary healthcare strengthening	Advocated integrated health systems
Jabbarpour and Raney (2017) [45]	Systematic review	Practice transformation	Improved cost and quality outcomes

### 6.3. Public Dental System Performance

Evidence from public dental systems shows that fragmentation, limited specialist availability, and inconsistent referral networks contribute to treatment delays and reduced quality of care [10, 11]. In contrast, integrated endodontic programs, particularly those incorporating workforce training, colocation, and performance monitoring, demonstrate increased productivity, improved service efficiency, and greater equity in care distribution [12].

### 6.4. Impact on Patient Outcomes

Studies assessing patient‐reported outcomes indicate that integrated endodontic care enhances satisfaction, reduces anxiety associated with treatment delays, and supports adherence to follow‐up appointments [9, 13]. Early intervention and coordinated management also translate into clinical benefits, including improved pulp vitality preservation, reduced postoperative discomfort, and better long‐term tooth survival [14–16]. Overall, these findings reinforce the value of integrating endodontic care within preventive oral health frameworks as part of comprehensive primary care delivery.

## 7. Barriers and Facilitators

The integration of endodontic services within preventive oral health systems is influenced by multiple interrelated barriers and facilitators operating at systemic, workforce, and technological levels.

### 7.1. Key Barriers

#### 7.1.1. Systemic Fragmentation

A lack of coordination between preventive and curative services remains a major barrier, resulting in fragmented care pathways and delayed treatment delivery [2, 7].

#### 7.1.2. Financial Constraints

Endodontic procedures are often inconsistently reimbursed or excluded from public health benefit packages, limiting accessibility, particularly in low‐resource settings [20, 21].

#### 7.1.3. Workforce Limitations

Shortages of trained endodontic specialists and enhanced‐skill practitioners restrict the feasibility of integration and increase reliance on overburdened primary care providers [11, 12].

#### 7.1.4. Technological Gaps

Limited access to digital tools such as electronic health records, digital radiography, and teledentistry reduces the ability to coordinate care efficiently and hampers communication across service levels [18, 24].

### 7.2. Facilitators

#### 7.2.1. Policy Support

National and international policy frameworks that incorporate oral health within universal health coverage provide a strong foundation for integrated endodontic service delivery [20, 21].

#### 7.2.2. Education

Curricula and continuing education programs emphasizing minimally invasive and prevention‐oriented endodontics enhance provider competence and facilitate integration [11, 15].

#### 7.2.3. Technology

AI‐driven diagnostics, digital radiography, and teledentistry platforms facilitate remote decision‐making, referral triage, and collaborative care [18, 24].

#### 7.2.4. Leadership

Local clinical leadership, interprofessional collaboration, and system‐level champions play a critical role in driving adoption and scaling integrated models [4, 8].

## 8. Policy and Implementation Framework

Effective integration of endodontic services into preventive oral health care requires a multilevel, system‐wide approach, supported by policy, education, service delivery, and monitoring mechanisms. A structured implementation framework is essential to translate integration concepts into practice across diverse healthcare settings.

### 8.1. Policy Level

National oral health frameworks that incorporate endodontic care into essential benefit packages strengthen access and ensure continuity of services across primary and secondary care [19, 20, 22]. Embedding oral health within universal health coverage models provides structural support for referral coordination, early intervention, and equitable access to endodontic procedures. In addition, policy standardization of referral pathways and care protocols is critical to ensure consistency and reduce variability in service delivery.

### 8.2. Educational Level

Curricular reforms emphasizing prevention‐oriented endodontics, vital pulp therapy, early pulpal diagnosis, and minimally invasive strategies are essential for preparing a competent primary‐care workforce [11, 15]. Integration of endodontic competencies into undergraduate and continuing dental education enhances provider readiness and supports consistent and high‐quality service delivery. Ongoing competency assessment and structured training pathways are also necessary to maintain clinical standards within integrated models.

### 8.3. Service Delivery Level

Implementation requires standardized referral systems, digital tracking mechanisms, and clear protocols that guide coordination between general dentists, enhanced‐skill practitioners, and specialist endodontists [4, 5]. Digital platforms, including electronic health records, teledentistry, and AI‐supported triage tools, facilitate referral validation, streamline clinical decision‐making, and enable monitoring of treatment progress across levels of care [18]. Effective service delivery models should also incorporate clear case‐selection criteria to ensure appropriate allocation of cases between primary and specialist care.

### 8.4. Monitoring and Evaluation Level

Monitoring frameworks should include measurable indicators such as [20]:

• tooth preservation rate,
• referral completion rate,
• timeliness of care,
• complications avoided,
• patient‐reported outcomes.

These indicators enable evaluation of integration effectiveness and support continuous quality improvement within healthcare systems. International frameworks such as the FDI White Paper on Endodontic Care and the AAE NCRDSCB Report provide guidance for scaling integrated endodontic programs across diverse health systems [2, 6]. These frameworks emphasize the importance of aligned policy support, robust referral networks, enhanced workforce training, and evidence‐based clinical pathways.

## 9. Future Directions

Future research should prioritize robust evaluation of the cost‐effectiveness of integrated endodontic care models within preventive oral health systems to determine their long‐term value and feasibility [1, 23]. Additional priorities include standardized assessment of long‐term clinical outcomes such as tooth survival rates, postoperative pain, and durability of vital pulp therapies to better understand the sustained benefits of integration.

Advancements in artificial intelligence, digital diagnostics, and tele‐endodontic platforms warrant further investigation, particularly regarding their capacity to enhance screening accuracy and streamline referral pathways [18]. These technologies have the potential to enhance the early identification of pulpal involvement during routine preventive visits and improve communication between general dentists and specialists.

**Figure 4:**
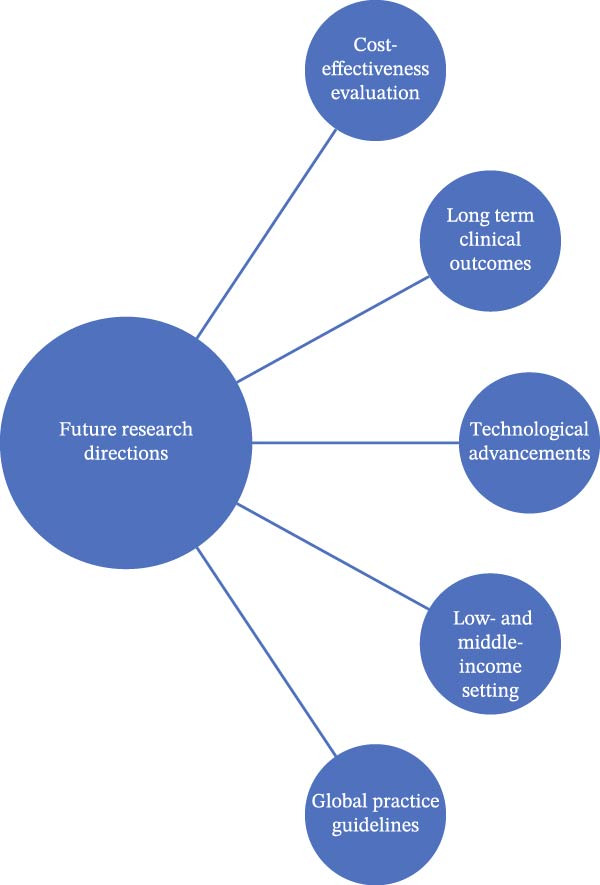
Exploring future directions in integrated endodontic care.

Further research is needed to explore the feasibility and adaptability of integrated endodontic care models in low‐ and middle‐income settings, where workforce shortages and infrastructural limitations present unique challenges [1, 3, 5]. Pilot implementation studies in these regions can provide insight into scalability, workforce adaptation, and the potential to reduce oral health disparities. Future work should also focus on developing standardized clinical guidelines for preventive‐endodontic triage, referral criteria, and comanagement protocols [6, 22]. Such guidelines would support harmonized decision‐making, improve continuity of care, and facilitate adoption of integrated approaches across diverse health‐system contexts. (Figure 4).

## 10. Discussion

The incorporation of endodontic services into preventive oral health programs represents a significant step toward improving care continuity, facilitating earlier diagnosis, and reducing the overall burden of pulpal and periapical conditions. Untreated dental caries and traumatic dental injuries remain major drivers of endodontic disease globally, particularly in low‐ and middle‐income regions where access to specialist services is limited [1, 3].

While preventive programs have traditionally focused on caries control and community‐based oral health promotion, they often lack structured assessment of pulpal status, resulting in missed opportunities for timely intervention [4, 5].

Integrating systematic endodontic screening and well‐defined referral pathways into preventive frameworks offer a practical approach to addressing this gap. Models described in the literature, ranging from referral‐based systems to colocated and digitally integrated care pathways, demonstrate improvements in treatment uptake and continuity of care [7, 8].

For example, initiatives such as the United Kingdom’s “Dentists with Enhanced Skills” programs have been associated with reduced waiting times and improved efficiency within public dental services [9, 10].

Advances in technology have further enhanced the feasibility of integrating preventive and endodontic care. Digital radiography, electronic health records, and teledentistry platforms facilitate early detection of pulpal pathology and enhance communication between providers [11, 14].

Advances in vital pulp therapy, regenerative endodontics, and minimally invasive instrumentation further align with prevention‐oriented approaches and support long‐term tooth preservation [12, 13]. Collectively, these developments expand the scope of endodontic care within primary settings and improve clinical outcomes.

### 10.1. Critical Appraisal

Despite these advancements, several challenges continue to limit widespread implementation. System‐level fragmentation, inconsistent reimbursement structures, and inadequate digital infrastructure continue to hinder effective coordination and scalability [4, 14].

Workforce limitations, lack of standardized training pathways, and challenges in ensuring specialist support further complicate implementation [15, 16]. Patient‐related factors, including dental anxiety, limited awareness of disease progression, and difficulty navigating referral systems, also negatively affect treatment uptake [17].

Importantly, the current evidence base is largely derived from observational and descriptive studies, with limited standardized outcome measures and insufficient long‐term data, restricting the ability to generalize findings across healthcare systems.

### 10.2. Policy and System Interpretation

Overcoming these barriers requires coordinated policy and system‐level interventions. Key strategies include the inclusion of endodontic services within essential benefit packages, the development of standardized referral protocols, and investment in digital infrastructure [16, 18, 19].

In practical application, the proposed integration model may function through a stepwise pathway beginning with preventive screening and early pulpal assessment at the primary‐care level, followed by standardized triage, referral coordination, and selective involvement of enhanced‐skill practitioners or specialist endodontists, depending on case complexity. Digital platforms and shared electronic records can support communication across these stages, while monitoring indicators such as referral completion and tooth preservation rates can facilitate continuous quality improvement. This framework allows flexibility for adaptation across healthcare systems with differing workforce and infrastructure capacities.

Workforce strengthening through targeted training, role expansion, and continuing education is essential to enhance service capacity. The incorporation of measurable performance indicators such as referral completion rates, tooth survival outcomes, and timeliness of care can further improve accountability and system performance [20].

Overall, the available evidence supports integration as a means of improving tooth retention, reducing emergency interventions, enhancing patient experiences, and strengthening public dental systems [20]. However, the effectiveness of integration models is highly context‐dependent, varying based on healthcare infrastructure, workforce availability, and policy support. This highlights the need for adaptable, context‐specific implementation strategies rather than a uniform approach.

Aligning preventive strategies with endodontic care through structured referral systems, skilled workforce deployment, and digital coordination can facilitate the transition toward patient‐centered, minimally invasive, and sustainable models of care. Future research should prioritize cost‐effectiveness analyses, standardized outcome measures, and evaluation of long‐term clinical impact across diverse healthcare settings.

## 11. Conclusion

Integrating endodontic services within preventive oral health programs represents a strategic advancement toward comprehensive, patient‐centered care. Available evidence indicates that early diagnosis, structured referral systems, and preventive endodontic approaches can improve clinical outcomes, reduce emergency visits, and enhance tooth preservation [1–6, 9].

Embedding pulp‐focused assessment, vital pulp therapy, and minimally invasive interventions within primary care can help address existing service gaps and reduce treatment delays, particularly in underserved populations [7–11]. Advances in digital technologies, including radiography, electronic health records, teledentistry, and artificial intelligence, further support integration by enabling early detection and improving treatment planning [16–18].

However, sustainable implementation depends on supportive policy frameworks, workforce development, investment in digital infrastructure, and incorporation of endodontic competencies into dental education [20–22]. Standardized referral pathways, enhanced‐skill practitioner models, and coordinated interprofessional systems are essential to ensure efficiency and equity in service delivery.

Overall, integration represents a system‐level transformation that aligns preventive dentistry and endodontics toward the shared goals of disease prevention, early intervention, and long‐term oral health preservation. Future research should focus on cost‐effectiveness, long‐term clinical outcomes, and the scalability of integration models across diverse healthcare settings.

## Author Contributions

**Conceptualization:** Chitharanjan M. Shetty and Shreya Hegde. Methodology: Derek Shaji Pious and Maria Anna Geevarghis. Literature search and data curation: Derek Shaji Pious, Rashi Shroff, and Gurmeen Kaur. Writing – original draft: Derek Shaji Pious and Maria Anna Geevarghis. Writing – review and editing: Chitharanjan M. Shetty and Shreya Hegde. Supervision: Shreya Hegde. Project administration: Chitharanjan M. Shetty.

## Funding

This study did not receive any specific funding.

## Disclosure

All authors have read and approved the final version of the manuscript. The corresponding author had full access to all of the data in this study and takes complete responsibility for the integrity of the data and the accuracy of the data analysis. All references cited in this manuscript were checked for retractions and corrections using PubMed and other available databases. No retracted articles were identified. Where applicable, any corrections to the cited articles were reviewed and were not found to affect the interpretation or conclusions of this manuscript.

## Ethics Statement

This study is a narrative review based exclusively on previously published literature and publicly available data. It does not involve human participants, patient records, or identifiable personal information. Therefore, ethical approval and informed consent were not required, in accordance with institutional policies and international ethical guidelines for biomedical research.

## Conflicts of Interest

The authors declare no conflicts of interest.

## Data Availability

The authors confirm that the data supporting the findings of this study are available within the article and/or its referenced sources.
